# Proteomic Analysis of Liver from Human Lipoprotein(a) Transgenic Mice Shows an Oxidative Stress and Lipid Export Response

**DOI:** 10.1155/2018/4963942

**Published:** 2018-11-25

**Authors:** Euan J. Rodger, Carolyn M. Porteous, Gregory T. Jones, Michael Legge, Torsten Kleffmann, Sally P. A. McCormick

**Affiliations:** ^1^Department of Pathology, Dunedin School of Medicine, University of Otago, P.O. Box 56, Dunedin 9054, New Zealand; ^2^Maurice Wilkins Centre for Molecular Biodiscovery, Private Bag 92019, Auckland 1142, New Zealand; ^3^Department of Biochemistry, School of Biomedical Sciences, University of Otago, P.O. Box 56, Dunedin 9054, New Zealand; ^4^Department of Surgical Sciences, Dunedin School of Medicine, University of Otago, P.O. Box 56, Dunedin 9054, New Zealand; ^5^Centre for Protein Research, University of Otago, P.O. Box 56, Dunedin 9054, New Zealand

## Abstract

**Background:**

Mouse models of hypercholesterolaemia have been used to identify arterial proteins involved in atherosclerosis. As the liver is extremely sensitive to dyslipidemia, one might expect major changes in the abundance of liver proteins in these models even before atherosclerosis develops.

**Methods:**

Lipid levels were measured and a proteomic approach was used to quantify proteins in the livers of mice with an elevated low-density lipoprotein (LDL) and the presence of lipoprotein(a) [Lp(a)] but no atherosclerosis.

**Results:**

The livers of Lp(a) mice showed an increased triglyceride but reduced phospholipid and oxidised lipid content. Two-dimensional gel electrophoresis and mass spectrometry analysis identified 24 liver proteins with significantly increased abundance in Lp(a) mice (*P*<0.05). A bioinformatic analysis of the 24 proteins showed the major effect was that of an enhanced antioxidant and lipid efflux response with significant increases in antioxidant (Park7, Gpx1, Prdx6, and Sod1) and lipid metabolism proteins (Fabp4, Acaa2, apoA4, and ApoA1). Interestingly, human liver cells treated with Lp(a) showed significant increases in Gpx1 and Prdx6 but not Sod1 or Park7.

**Conclusions:**

The presence of human LDL and Lp(a) in mice promotes an enhanced flux of lipids into the liver which elicits an antioxidant and lipid export response before the onset of atherosclerosis. The antioxidant response can be reproduced in human liver cells treated with Lp(a).

## 1. Introduction

Mouse models of hypercholesterolaemia are widely used to study atherosclerosis. The two most widely used models are the apolipoprotein E deficient (ApoE-/-) mouse [1] with elevated levels of cholesterol and triglyceride due to defective remnant lipoprotein clearance [2] and the low-density lipoprotein receptor deficient (LDLR-/-) mouse with elevated cholesterol due to defective low-density lipoprotein (LDL) uptake. Both have served as a base for genetic, pharmacologic, and dietary interventions to establish the effects on atherosclerosis development [3]. A third model, the lipoprotein(a) [Lp(a)] transgenic mouse [4], exhibits a milder hypercholesterolemia driven by expression of human apolipoprotein B which elevates LDL levels and the presence of Lp(a) in the circulation due to expression of human apolipoprotein(a) [apo(a)]. Compared to the apoE-/- and LDLR-/- mice, the Lp(a) mice are slow to develop atherosclerosis [5] but are relevant to the human situation due to the elevated levels of human LDL and presence of Lp(a), both of which have been well established as important cardiovascular risk factors in humans [6, 7].

The hypercholesterolaemic mouse models have been subject to transcriptomic and proteomic approaches to investigate changes in arterial genes and proteins related to atherosclerosis development [8–10]. A transcriptomic study of LDLR-/- mice documented an inability to mount an antioxidant response as being central to the development of atherosclerosis [8]. A 2D-gel-based proteomic study of apoE-/- mice has shown that impairments in lipid and energy metabolism, combined with oxidative stress and inflammation, preceded lesion development [9]. In addition, a 2D-gel-based proteomic study of Lp(a) mice also showed that changes in lipid and energy metabolism and redox genes preceded lesion development albeit with fewer changes in a different subset of proteins compared to the apoE-/- mice, indicating that different plasma lipid profiles invoke different changes in the same tissue [10].

The liver has a central role in lipid metabolism and it synthesizes many of the proteins implicated in atherosclerosis. It is extremely sensitive to altered lipid flux driven by dyslipidemias [11] and prolonged alteration in lipid flux can lead to hepatic steatosis in conjunction with atherosclerosis, as can be seen in the apoE-/- model with age [12]. Surprisingly few proteomic studies of dyslipidemia mouse models have looked at changes in liver proteins. One study performed a proteomic analysis of isolated liver mitochondria from apoE-/- mice [13] and showed an altered abundance of proteins related to lipid metabolism, apoptosis, and antioxidant and detoxifying mechanisms. Here, we investigated changes in liver protein abundances in the Lp(a) mice preceding atherosclerosis. We hypothesized that the Lp(a) mice would show differential expression of liver proteins due to the altered lipid flux posed to the liver by the elevated human LDL and presence of Lp(a).

## 2. Materials and Methods

### 2.1. Mice

The mice (*mus musculus*) used here were the same wildtype C57BL/6 mice and Lp(a) transgenic mice used previously to investigate arterial protein expression [10]. The C57BL/6 background in both lines was confirmed by genotyping with C57BL/6-specific markers provided by Saturn Biotech Limited, Perth, Australia. Mice were fed a chow diet (Ruakura 86 (5.2% fat) Sharps, Carterton, New Zealand) and housed in a specific pathogen-free animal facility with a 12-hour light/dark cycle. The livers were harvested from 20 Lp(a) transgenic and 20 wildtype female mice at the age of 30 weeks. The livers of a further 4 Lp(a) and 4 wildtype mice of the same sex and age were used to further validate results for one of the proteins identified from the proteomic analysis. All animal experimental procedures were approved by the Institutional Animal Use Ethics Committee.

### 2.2. Plasma Lipid Analysis

Whole blood was collected via cardiac puncture and transferred to EDTA microtubes. Plasma was isolated and stored at -80°C. The distribution of cholesterol and triglyceride amongst plasma lipoproteins was analyzed by separation of pooled plasma samples by gel permeation chromatography (n=4 samples per pool) on a Superose 6HR 10/30 column from GE Healthcare Bio-Sciences (Uppsala, Sweden). Separated fractions were measured for cholesterol and triglycerides by enzymatic assay (Roche, Mannheim, Germany).

### 2.3. Hepatic Lipid Analysis

The livers from 12 Lp(a) transgenic mice and 12 wildtype mice were rinsed in phosphate-buffered saline (PBS) and stored at -80°C. Lipids were extracted from 500 mg biopsies of each liver following the method by Bartels* et al*. [14]. Tissue was homogenized in PBS containing a protease inhibitor cocktail (Roche) and lipids were extracted with chloroform/methanol and evaporated under nitrogen gas. Lipids were resuspended in isopropanol containing 1% Triton X-100. Cholesterol, triglyceride, and phospholipid levels in the lipid extracts were measured using enzymatic reagents (Roche). A fluorometric thiobarbituric acid reactive substances (TBARS) assay [15] was used to measure the concentration of aldehydes as a measure of oxidised lipids in the lipid extracts.

### 2.4. Proteomic Analysis

The remaining frozen liver sections from 12 Lp(a) transgenic and 12 wildtype mice were homogenized in Tri-reagent (Progenz, Auckland, New Zealand) containing 2% of a protease inhibitor cocktail (Roche) on ice. Proteins were precipitated with isopropanol and subsequently washed with ethanol and contaminants were removed using a 2D cleanup kit (GE Healthcare). The liver protein extracts were used for 2D PAGE proteomic analysis using the Mini-PROTEAN 2D electrophoresis system (Bio-Rad, Hercules, CA). Three replicate gels were run for each mouse. Following electrophoresis, all gels were stained with colloidal Coomassie brilliant blue and scanned with a calibrated densitometer (ImageScanner, GE Healthcare). The 2D PAGE images were analyzed with the ImageMaster 2D platinum software (GE Healthcare) and spots exhibiting a statistical difference (p<0.05) were excised for identification by mass spectrometry.  Text S1 in the Supporting Information contains a detailed description of the proteomic analysis including the averaging and normalisation of protein spots for statistical analysis along with mass spectrometry methods.

### 2.5. Western Blotting

Western blots were used to validate the comparative 2D proteomic analysis for the antioxidant enzymes. Following 2D PAGE, liver proteins were transferred onto nitrocellulose membrane using the PROTEAN Trans-Blot transfer system (Bio-Rad). Membranes were incubated overnight with primary polyclonal antibodies (all from Abcam, Cambridge, UK) against Gpx1 (ab22604), Prdx6 (ab59543), and Sod1 (ab13498). Membranes were then incubated with HRP-conjugated anti-rabbit IgG (Thermo Fisher Scientific, Rockford, IL) or HRP-conjugated anti-goat IgG (Santa Cruz Biotechnology, Dallas, TX) for 2 hours at room temperature. For the Park7 protein, liver proteins were separated by SDS PAGE and transferred onto nitrocellulose membrane and the membrane was probed with an anti-Park7 antibody (ab18257). Membranes were incubated in ECL reagent and exposed under standard sensitivity on a LAS-3000 luminescent image analyzer (Fujifilm Corporation, Tokyo, Japan). Park7 protein levels were quantified by densitometry using Image Studio Lite (LI-COR Biosciences, Inc.) after normalisation to actin.

### 2.6. Lipoprotein Purification

Lp(a) was isolated from human plasma as previously described using a combination of density ultracentrifugation and size exclusion chromatography [16]. LDL was isolated from human plasma by density ultracentrifugation [17]. Purified lipoproteins were analysed by lipoprotein electrophoresis and western blotting and protein concentrations quantified using the Qubit protein assay kit (Thermo Fisher Scientific, Waltham, MA).

### 2.7. Cell Culture Treatments

Human hepatocellular carcinoma (HepG2) cells (American Type Culture Collection, Manassas, VA) were maintained in Advanced Dulbecco's Modified Eagle Medium (DMEM) supplemented with 10% fetal bovine serum (Bio International, Auckland, New Zealand), 2 mM L-glutamine, 0.25 *μ*g/mL amphotericin B, 100 U/mL penicillin, and 100 *μ*g/mL streptomycin (Life Technologies) at 37°C in a humidified environment with 5% CO_2_. HepG2 cells were seeded at 5×10^5^ cells/mL. 24 hours after seeding, cells were treated with 5 *μ*g/ml of either purified Lp(a) or LDL for 6 hours at 37°C. Cell lysates were harvested and 40 *μ*g of cell lysate protein was subject to western blot analysis with anti-Gpx1 (ab108427, Abcam), anti-Prdx6 (ab16947 Abcam), and anti-SOD (ab13498 Abcam) antibodies. An anti-actin antibody (Novus NB100-74340) was used as a loading control. Protein levels were quantified by densitometry using Image Studio Lite (LI-COR Biosciences, Inc) after normalisation to actin.

### 2.8. Statistics and Bioinformatics

Statistical analyses were performed using GraphPad Prism (GraphPad, San Diego, CA). A nonpaired Student's* t*-test was used to test for significant differences in mean hepatic lipid levels, the mean normalized spot volumes obtained from 2D PAGE analysis of the Lp(a) transgenic versus wildtype mice, the mean Park7/actin ratio, and the mean protein band intensity from lipoprotein-treated HepG2 cells. A difference with* P*<0.05 was considered significant. The AmiGO search tool was used to screen the gene ontology (GO) database to annotate the functional relevance of proteins identified as being significantly different in relative abundance between Lp(a) and wildtype mice. Protein network analysis was performed by uploading the list of proteins to the online STRING database tool (http://string-db.org) [18] and selecting “experiments”, “databases”, and “text mining” as interaction sources.

## 3. Results

### 3.1. Plasma Lipid Levels

The plasma lipid levels of the Lp(a) mice used for this study have been previously reported and show elevated cholesterol, triglyceride, and phospholipid levels as a result of elevated LDL and HDL cholesterol and the presence of Lp(a) [10]. Separation of the plasma lipoproteins by fast liquid protein chromatography showed the LDL to be triglyceride-rich (see Supporting Information, Figure S1).

### 3.2. Liver Lipid Levels in Lp(a) Transgenic Mice

**Figure 1:**
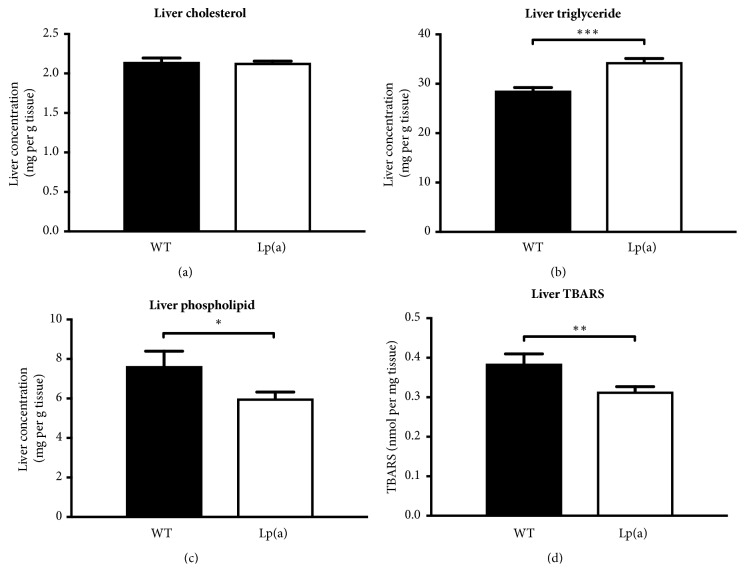
**Lipid levels in the livers of wildtype and Lp(a) mice. **Lipids were extracted from the livers of wildtype (WT) and Lp(a) mice (n=12). Cholesterol concentrations (a), triglyceride concentrations (b), phospholipid concentrations (c), and thiobarbituric acid-reactive substances (TBARS) concentrations (d). Data represented as mean ± SEM. ^*∗*^P<0.05, ^*∗∗*^P<0.01, and ^*∗∗∗*^P<0.001 versus wildtype.

The livers of Lp(a) transgenic mice showed similar cholesterol (Figure 1(a)) but significantly higher triglyceride levels than wildtype mice (34.4 ± 1.7 versus 28.6 ± 1.5 mg/g, P<0.001, Figure 1(b)). Phospholipid levels were significantly lower in the Lp(a) transgenic mice (6.0 ± 0.3 versus 7.6 ± 0.8 mg/g, P<0.05, Figure 1(c)) as were the levels of thiobarbituric acid-reactive substances (TBARS), a measure of lipid oxidation products (0.31 ± 0.03 versus 0.38 ± 0.06 nmol/mg, P < 0.05, Figure 1(d)).

### 3.3. Liver Proteomics Analysis

**Figure 2:**
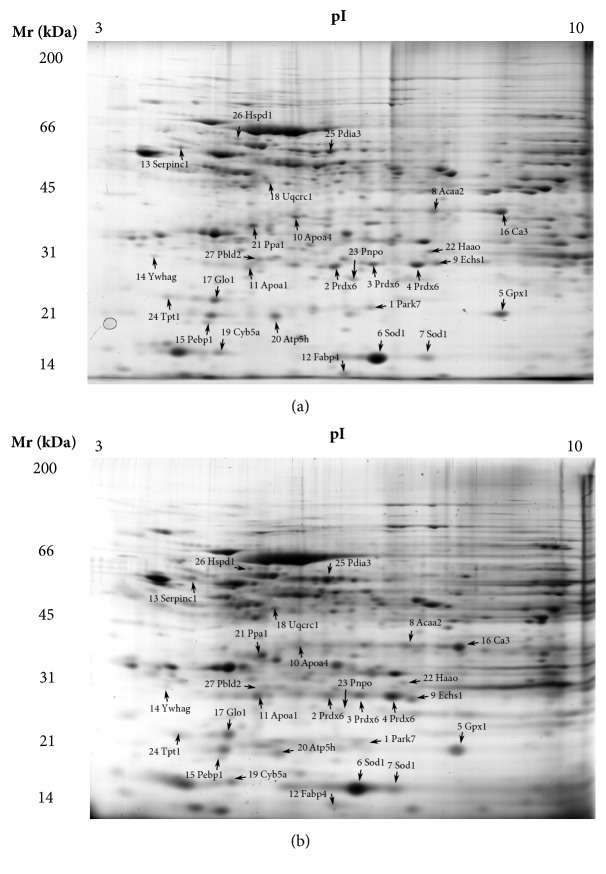
**Representative 2D PAGE images of proteins in the livers of wildtype and Lp(a) mice.** Liver protein extracts from 12 wildtype mice (a) and 12 Lp(a) mice (b) were separated by 2D PAGE in triplicate. Comparative analysis of equivalent spots between the averaged gels of the Lp(a) and wildtype mice identified 27 spots with significantly different intensities. Protein spots showing a significant difference in relative abundance (P<0.05) are indicated by their abbreviated protein names and are listed in Table 1.

**Figure 3:**
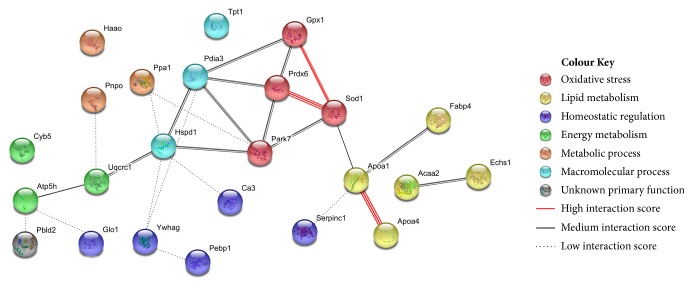
**STRING protein interaction network of proteins showing altered abundance in the livers of Lp(a) transgenic mice.** Functional interactions between the 24 proteins significantly different between the livers of Lp(a) mice (n = 12) and wildtype mice (n = 12) were predicted using the STRING database tool. The protein nodes are coloured according to their assigned functional grouping from GO analysis. The interaction edges (connecting lines) are depicted based on interaction confidence score: high (0.7), medium (0.4), or low (0.15).

Representative 2D PAGE images from the livers of Lp(a) and wildtype mice on which the proteomic analysis was based are shown in Figures 2(a) and 2(b) (replicate gels are shown in Figures S2 and S3). A total of 202 spots were detected within all 2D-gel replicates. A comparative analysis of equivalent spots between the averaged gels of the Lp(a) and wildtype mice (n =12 gels for both) identified 27 spots with significantly different intensities (25 increased and 2 decreased). The 27 spots, which were excised and identified by mass spectrometry, are shown on the 2D PAGE images (Figure 2) and listed in Tables 1 and S1. As two of the identified proteins, peroxiredoxin 6 (Prdx6) and superoxide dismutase 1 (Sod1), had multiple spots, this gave 24 unique protein identities. Interestingly, 14 of these proteins were on a list of proteins with functional relevance to CVD [19]. A gene ontology (GO) analysis to group the proteins into functional categories showed proteins involved in lipid metabolism and oxidative stress functions were prominent (Table 1). A protein interaction analysis using the STRING database tool (Figure 3) predicted a strong cluster of oxidative stress proteins (red nodes: Park7, Gpx1, Prdx6, and Sod1) as well as a lipid metabolism cluster (yellow nodes: Acaa2, Echs1, Apoa4, ApoA1, and Fapb4A). With respect to oxidative stress, several antioxidant enzymes showed a significant > 2-fold increase in abundance in the Lp(a) transgenic mice compared to wildtype mice including a 2.9-fold increase in glutathione peroxidase 1 (Gpx1), a 3.2-fold increase in superoxide dismutase 1 (Sod1), and a 2.2- and 3.4-fold increase in two forms of peroxiredoxin 6 (Prdx6). With respect to lipid metabolism, there was a 2.1-fold increase in fatty acid binding protein 4 (Fabp4) and a 2.8-fold increase in the beta-oxidation enzyme 3-ketoacyl-CoA thiolase (Acaa2). The apolipoproteins A1 (Apoa1) and A4 (Apoa4) involved in cholesterol efflux were significantly increased (4-fold and 2.7-fold, resp.).

### 3.4. Analysis of Antioxidant Response Proteins by Western Blot Analysis

**Figure 4:**
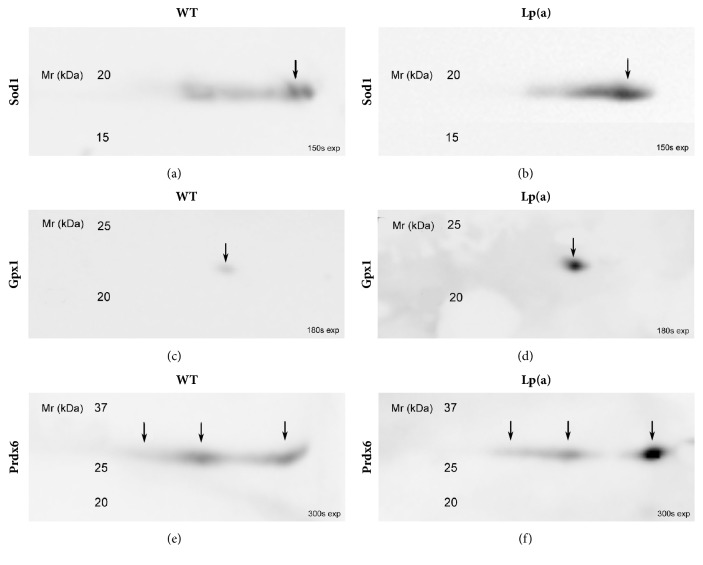
**Representative 2D-PAGE Western blot images of antioxidant proteins in wildtype and Lp(a) mice.** Western blots were used to validate the comparative proteomic analysis between the Lp(a) and wildtype mice for antioxidant proteins. Pooled liver protein extracts (n = 12) were separated by 2D PAGE in triplicate and transferred onto nitrocellulose membrane. Each membrane was probed with one of the following primary antibodies: Sod1 (a, b), Gpx1 (c, d), or Prdx6 (e, f). The proteins of interest were detected with an HRP-conjugated secondary antibody and imaged on a LAS-3000 luminescent analyzer. Arrows indicate the protein spots that correspond to those identified by 2D-PAGE analysis.

**Figure 5:**
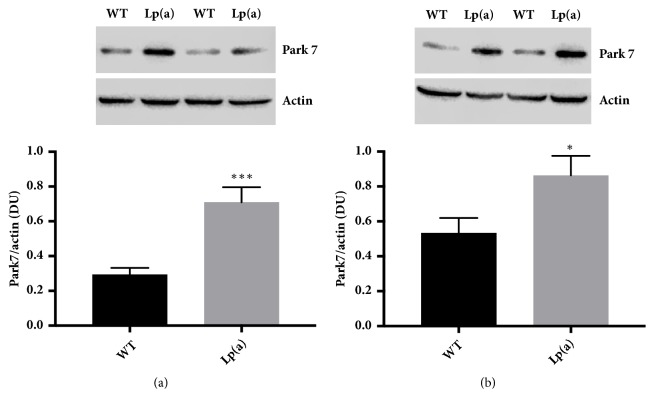
**Increased relative abundance of Park7 in Lp(a) mice**. Western blots were used to validate the comparative proteomic analysis between the Lp(a) and wildtype mice for the Park7 protein. Pooled liver protein extracts (n = 4 livers per pool) were separated by SDS PAGE in multiple replicates (n = 7) and transferred onto nitrocellulose membrane. Membranes were probed with an anti-Park7 antibody using an anti-actin antibody as a loading control. Liver protein extracts were the same as those used for Figure 4(a) as well as fresh liver protein extracts from new mice of the same age, sex, and genotype (b). Representative blots showing two of the replicates for each pooled liver protein extract are shown. Park7 protein levels were normalized against actin and expressed as a ratio in densitometry units (DU). Data is represented as mean ± SEM for the pooled samples run in septuplicate. *∗*P<0.05, ⁎⁎⁎P<0.001, versus wildtype.

Western blot analysis of 2D PAGE gels for the antioxidant enzymes Sod1, Gpx1, and Prdx6 (Figure 4) confirmed increased levels of these proteins in the livers of Lp(a) mice compared to wildtype mice, with multiple forms being apparent for Prdx6. Western blot analysis of Park7 confirmed an increased abundance of the protein in the livers of Lp(a) compared to wildtype mice (Figure 5(a)). This result was replicated in another set of liver samples from Lp(a) and wildtype mice of the same sex and age (Figure 5(b)).

### 3.5. Upregulation of Gpx1 and Prdx by Lp(a) in Human HepG2 Cells

**Figure 6:**
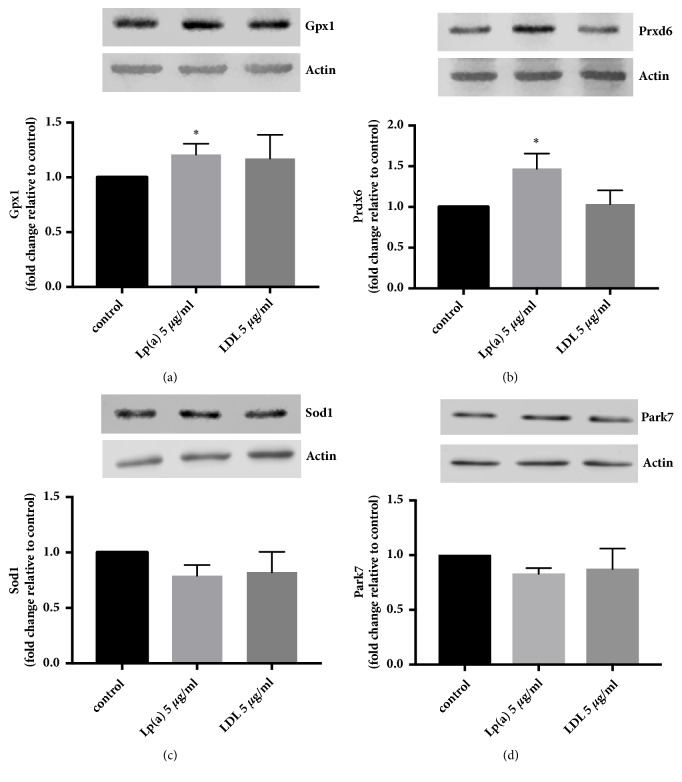
**Lp(a) upregulates GPx1 and Prdx6 expression in human HepG2 cells. **HepG2 cells were treated with 5 *μ*g/mL of Lp(a) or LDL for 6 hours at 37°C. Western blots of cell lysates were performed with an anti-Gpx1 antibody (a), an anti-Prdx6 antibody (b), an anti-Sod1 antibody (c), and an anti-Park7 antibody (d) using an anti-actin antibody as a loading control. Representative blots are shown. Protein levels were normalized against actin and expressed relative to that of untreated cells. Results are expressed as mean ± SEM for pooled triplicate incubations run in quadruplicate. *∗*P<0.05, relative to untreated HepG2 cells.

To investigate whether Lp(a) or LDL could elicit a direct effect* in vitro* on antioxidant proteins in the livers of Lp(a) transgenic mice, human HepG2 liver cells were treated with the two purified lipoproteins. Treatment with Lp(a) significantly increased the expression of GPx1 and Prdx6, but not Sod1 or Park7 (Figures 6(a)–6(d)). Treatment with LDL showed no significant effect on any of the four proteins (Figures 6(a)–6(d)).

## 4. Discussion

This study aimed to establish the changes in hepatic proteins in the Lp(a) mouse model of hypercholesterolemia. This model constitutes a milder form of hypercholesterolaemia than the apoE-/- or LDLR-/- mice [1, 2] and presents the opportunity to study tissue responses before the onset of disease. A previous study of Lp(a) mice showed no signs of atherosclerosis in their arteries but significant changes in the abundance of several arterial proteins [10]. Others have shown that atherosclerosis does develop in these Lp(a) mice with aging [5] or fat feeding [20]. As the liver is sensitive to hyperlipidaemia and also synthesizes many proteins involved in atherosclerosis, it is of interest to investigate changes in hepatic protein abundances. The Lp(a) mouse is of particular relevance to humans, since it contains elevated levels of the two most commonly elevated plasma lipoproteins, LDL and Lp(a) [21].

Despite significantly elevated plasma cholesterol levels [10], the Lp(a) mice had no accumulation of cholesterol in the liver, presumably due to the liver's ability to tightly regulate cholesterol levels. The elevated hepatic triglyceride levels are likely due to the uptake of triglyceride-rich LDL and suggest an enhanced flux of fatty acids into the liver. The proteomic analysis of liver supported this with a significant increase in the fatty acid transporter, Fabp4, and two *β*-oxidation enzymes (Acaa2 and Echs1). An increase in *β*-oxidation might also underlie the increase in the Uqcrc1 and Atp5h subunits of the electron transport chain. This response was in contrast to that seen in the arteries of the Lp(a) mice where there was significant decrease in Fabp4 and electron transport chain proteins [10], presumably reflecting the different metabolic capacities of these two different tissue types.

An increase in *β*-oxidation and electron transport chain activity generates reactive oxygen species (ROS) [22]. An increase in ROS is known to upregulate Nrf2, a redox responsive transcription factor that enhances the transcription of endogenous antioxidant enzymes that protect against oxidative damage. ROS promote the oxidation of the Keap1 inhibitor protein which prevents Keap1 from binding and targeting Nrf2 for constitutive degradation via ubiquitination [23]. Interestingly, the proteomic analysis of Lp(a) mice livers showed an increase in Park7 which was further validated in another set of Lp(a) and wildtype mice. Park7 is an abundant multifunctional protein which acts as a redox sensor and molecular cochaperone in cellular responses to oxidative stress. It positively regulates the antioxidant response through competing with Keap1 for binding to Nrf2 and protecting Nrf2 from degradation [24]. The three antioxidant enzymes that displayed an increased abundance in the livers of Lp(a) mice, Sod1, Gpx1, and Prdx6, are all known targets for Nrf2. Sod1 is a superoxide scavenging enzyme [25], whereas Gpx1 and Prdx6 regulate the levels of peroxides and lipid peroxides [26, 27]. Interestingly, multiple forms of Prdx6 were apparent which is in keeping with a proteomic study identifying several modified forms of Prdx6 in mouse liver [28]. Upregulation of all three antioxidant enzymes is associated with enhanced protection against oxidative stress [29–31]. The increase in Gpx1 and Prdx6 correlates with the significant decrease in TBARS found in the livers of Lp(a) mice as both enzymes metabolise lipid hydroperoxides into lipid alcohols which can be metabolised via *β*-oxidation thus preventing their breakdown to aldehydes. In contrast, the arteries of the Lp(a) mice accumulate TBARS and only show an increased abundance of one antioxidant enzyme, Prdx4, [10] which, unlike Prdx6, is only active against peroxides but not lipid peroxides. These differences likely reflect the different capacities of tissues to handle oxidised lipids.

Our results contrast with studies of apoE-/- mice that showed a decrease in antioxidant capacity with significant decreases in antioxidant enzymes in both liver mitochondria [13] and arteries [9]. The Lp(a) mice used here were of similar age to the apoE-/- mice used in those studies but the hyperlipidaemia is much milder and the Lp(a) mice were clearly still at a point where they could mount a robust antioxidant response in the liver. Interestingly, a proteomic analysis of the livers of transgenic Apoc3 mice which display a hypertriglyceridaemic phenotype, but no atherosclerosis [32], also showed an upregulation of cytosolic antioxidant enzymes, albeit amongst a slightly different set of proteins to the Lp(a) mice, indicating that different lipoprotein profiles may elicit different responses with respect to the proteins that are regulated.

With respect to lipid metabolism, the proteomic data also indicated an increased abundance of proteins involved in lipid export. Compared to the livers of wildtype mice, the Lp(a) mice had significant increases in both Apoa1 and Apoa4 (Table 1). ApoA1 facilitates the export of cholesterol from cell membranes via its interaction with the ATP binding cassette 1 (ABCA1) cholesterol transporter protein to generate HDL [33]. Apoa4 is a component of HDL that has also been shown to promote cellular cholesterol efflux [34] as well as facilitating triglyceride export from the liver [35]. The increase of Apoa1 and Apoa4 could contribute to the increase in HDL levels seen in the Lp(a) mice and may partially underlie the lack of cholesterol accumulation in the livers of the Lp(a) mice. The elevated levels of HDL would be expected to be atheroprotective and therefore potentially modulate the effect of the LDL and Lp(a) risk factors in the Lp(a) mice.

Lp(a) is primarily cleared by the liver [36] via a number of receptors including the LDLR [37], scavenger receptor B1 (SR-B1) [16], and the plasminogen receptor KT (PlgRKT) [38]. We investigated whether Lp(a) alone could promote changes in the antioxidant enzymes, GPx1, Prdx6, and Sod1, by incubating purified Lp(a) with human hepatoma cells. The GPx1 and Prdx6 proteins were significantly increased with Lp(a) treatment (Figure 5(a)) but not with LDL (Figure 5(b)) and Sod1 levels were unchanged by either treatment. The increase in antioxidant enzymes in this acute exposure to Lp(a) did not appear to involve Park7. The antioxidant response to Lp(a) suggests that a specific component of Lp(a) may be promoting the GPx1 and Prdx6 response. Unique to Lp(a) is the apolipoprotein(a) protein which specifically binds oxidised phospholipids [39] allowing the Lp(a) particles to bind significantly more oxidised phospholipid molecules than LDL. It is tempting to speculate that the oxidised phospholipids in Lp(a) may promote their own detoxification via upregulating GPx1 and Prdx6; however, further experiments would be needed to specifically investigate this.

## 5. Conclusions

We have performed a proteomic analysis on the livers of human Lp(a) transgenic mice and shown an increased abundance in antioxidant and lipid export proteins. Our findings suggest that the initial response to mild hyperlipidaemia is to invoke protective mechanisms that reduce the accumulation of lipid and its oxidised products. This contrasts with mouse models of a similar age with more extreme hyperlipidaemia that have lost this protection mechanism and display atherosclerosis.

## Figures and Tables

**Table 1:** Proteins showing significant (*P*<0.05) differential expression in the livers of Lp(a) versus wildtype mice on a normal chow diet.

**2D Gel Spot Number**	**Identified Protein** **(Swiss-Prot Accession Number)**	**Fold Change** **∗**	**P value**	**Primary Function(s)**
**Oxidative Stress**				
1	Protein DJ-1 (Park7, Q99LX0)	2.3	< 0.05	Oxidative stress response
2	Peroxiredoxin 6 (Prdx6, O08709)	2.2	< 0.05	Peroxide metabolism
3	Peroxiredoxin 6 (Prdx6, O08709)	3.4	< 0.0001	Peroxide metabolism
4	Peroxiredoxin 6 (Prdx6, O08709)	1.9	< 0.05	Peroxide metabolism
5	Glutathione peroxidase 1 (Gpx1, Q5RJH8)	2.9	< 0.01	Peroxide metabolism
6	Superoxide dismutase 1 (Sod1, P08228)	1.9	< 0.05	Superoxide metabolism
7	Superoxide dismutase 1 (Sod1, P08228)	3.2	< 0.05	Superoxide metabolism
**Lipid Metabolism**				
8	3-ketoacyl-CoA thiolase (Acaa2, Q8JZR8)	2.8	< 0.05	Fatty acid oxidation
9	Enoyl-CoA hydratase (Echs1, Q8BH95)	-2.6	< 0.01	Fatty acid oxidation
10	Apolipoprotein A-IV (Apoa4, P06728)	2.7	< 0.01	Lipid transport
11	Apolipoprotein A-I (ApoA1, Q00623)	4.0	< 0.05	Lipid transport
12	Fatty acid-binding protein 4 (Fabp4, P04117)	2.1	< 0.05	Fatty acid transport
**Homeostatic Regulation**				
13	Antithrombin III (Serpinc1, P32261)	1.9	< 0.05	Blood coagulation
14	14-3-3 protein gamma (Ywhag, P61982)	2.2	< 0.05	Signal transduction
15	Phosphatidylethanolamine binding protein 1 (Pebp1, P70296)	1.6	< 0.001	Signal transduction
16	Carbonic anhydrase 3 (Ca3, P16015)	-1.9	< 0.01	pH homeostasis
17	Lactoylglutathione lyase (Glo1, Q9CPU0)	1.8	< 0.001	Methylglyoxal detoxification
**Energy Metabolism**				
18	Cytochrome b-cl complex subunit 1 (Uqcrc1, Q00896)	4.2	< 0.05	Electron transport
19	Cytochrome b5 (Cyb5a, P56395)	3.2	< 0.05	Electron transport
20	ATP synthase subunit d (Atp5h, Q9DCX2)	1.7	< 0.05	ATP synthesis
**Metabolic Process**				
21	Inorganic pyrophosphatase (Ppa1, Q9D819)	1.9	< 0.05	Phosphate metabolism
22	3-hydroxy anthranilate 3,4-dioxygenase (Haao, Q78JT3)	1.5	< 0.05	Quinolinic acid metabolism
23	Pyridoxine-5'phosphate oxidase (Pnpo, Q91XF0)	2.7	< 0.01	Pyridoxine biosynthesis
**Macromolecular Process**				
24	Translationally controlled tumor protein (Tpt1, P63028)	3.0	< 0.05	Microtubule stabilisation
25	Protein disulfide isomerase A3 (Pdia3, P27773)	1.6	< 0.05	Glycoprotein synthesis
26	60 kDa heat shock protein (Hspd1, P63038)	1.5	< 0.05	Protein folding
**Unknown Primary Function**				
27	Phenazine biosynthesis-like domain containing protein 2 (Pbld2, Q9CXN7)	1.9	< 0.01	Unknown

*∗* Fold change between Lp(a) versus wildtype mice. Positive number indicates an increased expression in the Lp(a) mice. Negative number indicates a decreased expression in the Lp(a) mice. Proteins are categorised by function as determined by gene ontology (GO) analysis.

## Data Availability

The datasets supporting the conclusions of this article are included within the article and its additional files.

## Supplementary Materials

Supplementary Materials Additional file 1: Detailed methods of proteomic and mass spectrometry analyses. Figure S1: Plasma triglyceride profiles of wildtype and Lp(a) mice. Figure S2: 2D-PAGE replicates of wildtype mouse liver. Figure S3: 2D-PAGE replicates of Lp(a) mouse liver. Table S1. Proteins identified by MALDI-TOF MS/MS from the livers of wildtype and Lp(a) mice.

## References

[1] Plump A. S., Smith J. D., Hayek T. (1992). Severe hypercholesterolemia and atherosclerosis in apolipoprotein E-deficient mice created by homologous recombination in ES cells. *Cell*.

[2] Ishibashi S., Goldstein J. L., Brown M. S., Herz J., Burns D. K. (1994). Massive xanthomatosis and atherosclerosis in cholesterol-fed low density lipoprotein receptor-negative mice.. *The Journal of Clinical Investigation*.

[3] Lee Y. T., Lin H. Y., Chan Y. W. (2017). Mouse models of atherosclerosis: a historical perspective and recent advances. *Lipids in Health and Disease*.

[4] Linton M. F., Farese R. V., Chiesa G. (1993). Transgenic mice expressing high plasma concentrations of human apolipoprotein B100 and lipoprotein(a). *The Journal of Clinical Investigation*.

[5] Berg K., Svindland A., Smith A. J. (2002). Spontaneous atherosclerosis in the proximal aorta of LPA transgenic mice on a normal diet. *Atherosclerosis*.

[6] Kamstrup P. R., Benn M., Tybjærg-Hansen A., Nordestgaard B. G. (2008). Extreme lipoprotein(a) levels and risk of myocardial infarction in the general population: the Copenhagen City Heart Study. *Circulation*.

[7] Erqou S., Kaptoge S., Perry P. L. (2009). Lipoprotein(a) concentration and the risk of coronary heart disease, stroke, and nonvascular mortality. *Journal of the American Medical Association*.

[8] Collins A. R., Lyon C. J., Xia X. (2009). Age-accelerated atherosclerosis correlates with failure to upregulate antioxidant genes. *Circulation Research*.

[9] Mayr M., Chung Y., Mayr U. (2005). Proteomic and Metabolomic Analyses of Atherosclerotic Vessels From Apolipoprotein E-Deficient Mice Reveal Alterations in Inflammation, Oxidative Stress, and Energy Metabolism. *Arteriosclerosis, Thrombosis, and Vascular Biology*.

[10] Rodger E. J., Suetani R. J., Jones G. T. (2012). Proteomic Analysis of Aortae from Human Lipoprotein(a) Transgenic Mice Shows an Early Metabolic Response Independent of Atherosclerosis. *PLoS ONE*.

[11] Browning J. D., Horton J. D. (2004). Molecular mediators of hepatic steatosis and liver injury. *The Journal of Clinical Investigation*.

[12] Bonomini F., Filippini F., Hayek T. (2010). Apolipoprotein E and its role in aging and survival. *Experimental Gerontology*.

[13] Suski M., Olszanecki R., Madej J. (2011). Proteomic analysis of changes in protein expression in liver mitochondria in apoE knockout mice. *Journal of Proteomics*.

[14] Bartels E. D., Lauritsen M., Nielsen L. B. (2002). Hepatic expression of microsomal triglyceride transfer protein and in vivo secretion of triglyceride-rich lipoproteins are increased in obese diabetic mice. *Diabetes*.

[15] Williamson K. S., Hensley K., Floyd R. A., Hensley K., Floyd R. A. (2003). Fluorometric and Colourimetric Assessment of Thiobarbituric Acid-Reactive Lipid Aldehydes in Biological Matrices. *Methods in Pharmacology and Toxicology: Methods in Biological Oxidative Stress*.

[16] Sharma M., Von Zychlinski-Kleffmann A., Porteous C. M., Jones G. T., Williams M. J., McCormick S. P. (2015). Lipoprotein (a) upregulates ABCA1 in liver cells via scavenger receptor-B1 through its oxidized phospholipids. *Journal of Lipid Research*.

[17] Havel R. J., Eder H. A., Bragdon J. H. (1955). The distribution and chemical composition of ultracentrifugally separated lipoproteins in human serum. *The Journal of Clinical Investigation*.

[18] Szklarczyk D., Franceschini A., Wyder S. (2015). STRING v10: protein-protein interaction networks, integrated over the tree of life. *Nucleic Acids Research*.

[19] Lovering R. C., Dimmer E., Khodiyar V. K. (2008). Cardiovascular GO annotation initiative year 1 report: Why cardiovascular GO?. *Proteomics*.

[20] Callow M. J., Verstuyft J., Tangirala R., Palinski W., Rubin E. M. (1995). Atherogenesis in transgenic mice with human apolipoprotein B and lipoprotein (a). *The Journal of Clinical Investigation*.

[21] Ellis K. L., Hooper A. J., Burnett J. R., Watts G. F. (2016). Progress in the care of common inherited atherogenic disorders of apolipoprotein B metabolism. *Nature Reviews Endocrinology*.

[22] Begriche K., Massart J., Robin M. A., Bonnet F., Fromenty B. (2013). Mitochondrial adaptations and dysfunctions in nonalcoholic fatty liver disease. *Hepatology*.

[23] Gupte A. A., Lyon C. J., Hsueh W. A. (2013). Nuclear factor (erythroid-derived 2)-like-2 factor (Nrf2), a key regulator of the antioxidant response to protect against atherosclerosis and nonalcoholic steatohepatitis. *Current Diabetes Reports*.

[24] Clements C. M., McNally R. S., Conti B. J., Mak T. W., Ting J. P.-Y. (2006). DJ-1, a cancer- and Parkinson's disease-associated protein, stabilizes the antioxidant transcriptional master regulator Nrf2. *Proceedings of the National Acadamy of Sciences of the United States of America*.

[25] Zelko I. N., Mariani T. J., Folz R. J. (2002). Superoxide dismutase multigene family: a comparison of the CuZn–SOD (SOD1), Mn-SOD (SOD2), and EC-SOD (SOD3) gene structures, evolution, and expression. *Free Radical Biology & Medicine*.

[26] Lubos E., Loscalzo J., Handy D. E. (2011). Glutathione peroxidase-1 in health and disease: from molecular mechanisms to therapeutic opportunities. *Antioxidants & Redox Signaling*.

[27] Fisher A. B. (2011). Peroxiredoxin 6: a bifunctional enzyme with glutathione peroxidase and phospholipase A(2) activities. *Antioxid Redox Signal*.

[28] Jeong J., Kim Y., Kyung Seong J., Lee K.-J. (2012). Comprehensive identification of novel post-translational modifications in cellular peroxiredoxin 6. *Proteomics*.

[29] Handy D. E., Lubos E., Yang Y. (2009). Glutathione peroxidase-1 regulates mitochondrial function to modulate redox-dependent cellular responses. *The Journal of Biological Chemistry*.

[30] Yoshida T., Maulik N., Engelman R. M., Ho Y.-S., Das D. K. (2000). Targeted disruption of the mouse sod I gene makes the hearts vulnerable to ischemic reperfusion injury. *Circulation Research*.

[31] Wang X., Phelan S. A., Forsman-Semb K. (2003). Mice with Targeted Mutation of Peroxiredoxin 6 Develop Normally but Are Susceptible to Oxidative Stress. *The Journal of Biological Chemistry*.

[32] Ehx G., Gérin S., Mathy G. (2014). Liver proteomic response to hypertriglyceridemia in human-apolipoprotein C-III transgenic mice at cellular and mitochondrial compartment levels. *Lipids in Health and Disease*.

[33] Oram J. F. (2003). HDL apolipoproteins and ABCA1 partners in the removal of excess cellular cholesterol. *Arteriosclerosis, Thrombosis, and Vascular Biology*.

[34] Fournier N., Atger V., Paul J.-L. (2000). Human ApoA-IV overexpression in transgenic mice induces camp-stimulated cholesterol efflux from J774 macrophages to whole serum. *Arteriosclerosis, Thrombosis, and Vascular Biology*.

[35] VerHague M. A., Cheng D., Weinberg R. B., Shelness G. S. (2013). Apolipoprotein A-IV Expression in Mouse Liver Enhances Triglyceride Secretion and Reduces Hepatic Lipid Content by Promoting Very Low Density Lipoprotein Particle Expansion. *Arteriosclerosis, Thrombosis, and Vascular Biology*.

[36] Cain W. J., Millar J. S., Himebauch A. S. (2005). Lipoprotein [a] is cleared from the plasma primarily by the liver in a process mediated by apolipoprotein [a]. *Journal of Lipid Research*.

[37] Hofmann S. L., Eaton D. L., Brown M. S., McConathy W. J., Goldstein J. L., Hammer R. E. (1990). Overexpression of human low density lipoprotein receptors leads to accelerated catabolism of Lp(a) lipoprotein in transgenic mice. *The Journal of Clinical Investigation*.

[38] Sharma M., Redpath G. M., Williams M. J. A., McCormick S. P. A. (2017). Recycling of apolipoprotein(a) after PlgRKT-mediated endocytosis of lipoprotein(a). *Circulation Research*.

[39] Bergmark C., Dewan A., Orsoni A. (2008). A novel function of lipoprotein [a] as a preferential carrier of oxidized phospholipids in human plasma. *Journal of Lipid Research*.

