# Supplementary material for: Proteomic Analysis of Liver from Human Lipoprotein(a) Transgenic Mice Shows an Oxidative Stress and Lipid Export Response
Source: Biomed Res Int. 2018 Nov 25;2018:4963942. doi: 10.1155/2018/4963942 (PMC6286786; doi:10.1155/2018/4963942)
Supplement: Supplementary Materials — Additional file 1: Detailed methods of proteomic and mass spectrometry analyses. Figure S1: Plasma triglyceride profiles of wildtype and Lp(a) mice. Figure S2: 2D-PAGE replicates of wildtype mouse liver. Figure S3: 2D-PAGE replicates of Lp(a) mouse liver. Table S1. Proteins identified by MALDI-TOF MS/MS from the livers of wildtype and Lp(a) mice. [file 4963942.f1.pdf]

Rodger et al. Proteomic analysis of liver from human lipoprotein(a) transgenic mice shows an oxidative stress and lipid export response

Additional file 1

Proteomic Analysis

Frozen liver sections from 12 Lp(a) transgenic and 12 wildtype mice were homogenized in Tri-reagent (Progenz, Auckland, New Zealand) containing 2% protease inhibitor cocktail (Roche) on ice. Proteins were precipitated with isopropanol and subsequently washed with ethanol and contaminants removed using a 2D clean-up kit (GE Healthcare). Liver proteins were resolubilised in 7M urea, 2M thiourea, 2% protease inhibitor cocktail, 1% ASB-14, 1% DTT, and 0.5% carrier ampholytes pH 3-10. The concentration of solubilised proteins was determined using the Ettan 2D Quant kit (GE Healthcare). For 2-D PAGE, 100 μg of protein was applied to a 75 mm IEF vertical tube gel (pH gradient, 3.5-10, nonlinear). The gels were focused for 7.4kVh at 20°C in a Mini-PROTEAN 2D electrophoresis system (Bio-Rad Laboratories). Following isoelectric focusing, the tube gels were equilibrated and then mounted onto 12.5% polyacrylamide gels in a Mini-PROTEAN III electrophoresis system (Bio-Rad Laboratories). Electrophoresis in the second dimension was performed at 100V until the dye-front reached the bottom of the gel. Following electrophoresis, gels were fixed in 20% methanol, 1.3% orthophosphoric acid and proteins then visualised by staining with colloidal Coomassie brilliant blue [1]. Stained gels were scanned with a calibrated densitometer (ImageScanner, GE Healthcare). The raw 2-D PAGE images were analysed with ImageMaster 2D platinum software (GE Healthcare). Raw spot volumes were log transformed and normalized by zero-centering through median subtraction before being subject to statistical analysis [2]. Spots exhibiting a statistical difference ($p<0.05$) were excised for identification by mass spectrometry.

Mass Spectrometry

Excised protein spots were digested with modified trypsin (Roche) as previously described [3]. Peptides were recovered by sequential extraction in 5% TFA and 5% acetronitrile and desalted on Zip tips (Millipore). Samples were analysed on a 4800 MALDI-TOF/TOF MS analyzer (ABSciex, Foster City, Ca) in positive ion reflector

mode with alpha cyano-4-hydroxycinnamic acid (CHCA) as the matrix. The 15-20 strongest precursor ions of each spot were used for MS/MS collision-induced dissociation analysis. The resulting spectra were processed through the GPS Explorer software (ABSciex, Foster City, Ca) and searched against the SwissProt database using the MASCOT search engine. The search allowed for up to 4 missed cleavage sites, a mass tolerance threshold of 75 ppm and a maximum fragment mass error of 0.4 Da. Cysteine carbamidomethylation and methionine oxidation were selected as variable modifications. Individual MS/MS peptide ion scores with a confidence interval calculated by the GPS Explorer software of >95% were accepted as significant. In some cases, multiple identifications for one protein spot were gained due to the search identifying homologues of the mouse protein.

References

1. Neuhoff V, Arold N, Taube D, Ehrhardt W: Improved staining of proteins in polyacrylamide gels including isoelectric focusing gels with clear background at nanogram sensitivity using Coomassie Brilliant Blue G-250 and R-250. *Electrophoresis* 1988, 9(6):255-262.
2. Meleth S, Deshane J, Kim H: The case for well-conducted experiments to validate statistical protocols for 2D gels: different pre-processing = different lists of significant proteins. *BMC Biotechnol* 2005, 5:7.
3. Shevchenko A, Jensen ON, Podtelejnikov AV, Sagliocco F, Wilm M, Vorm O, Mortensen P, Boucherie H, Mann M: Linking genome and proteome by mass spectrometry: large-scale identification of yeast proteins from two dimensional gels. *Proc Natl Acad Sci U S A* 1996, 93(25):14440-14445.

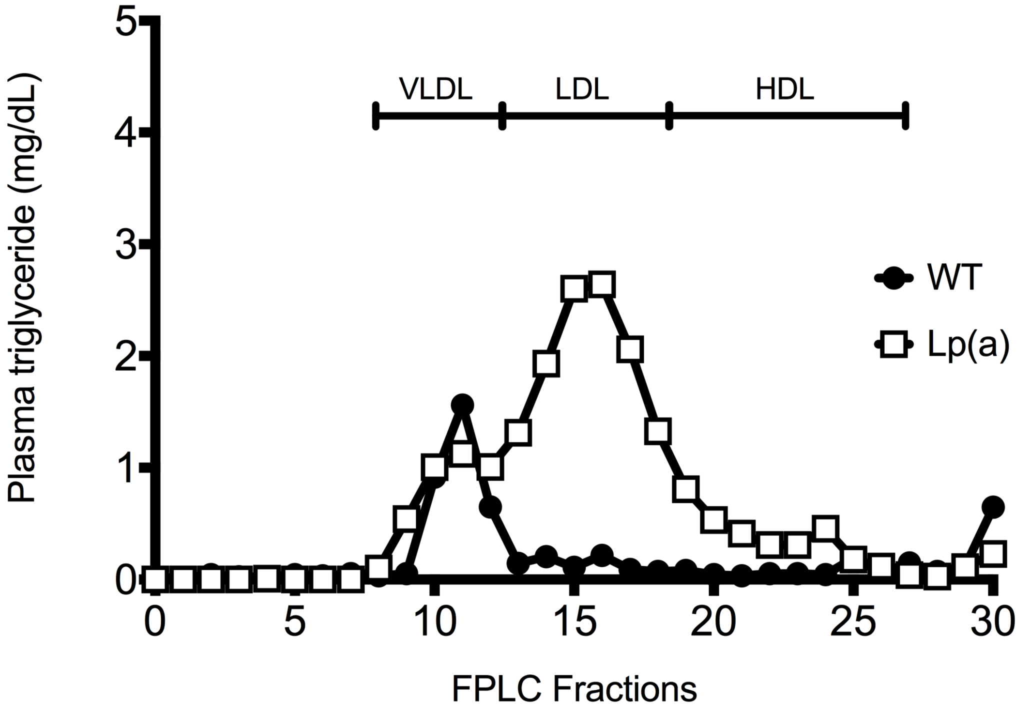

Figure S1. Plasma lipid profiles of wildtype and Lp(a) mice. Plasma lipoproteins from wildtype and Lp(a) mice (n=20) were separated by gel permeation chromatography and the triglyceride content of each fraction measured. The Lp(a) mice had TG-rich LDL particles, whereas the plasma TG in the wildtype mice was largely all in the VLDL.

Figure S2. 2D-PAGE replicates of wildtype mouse liver

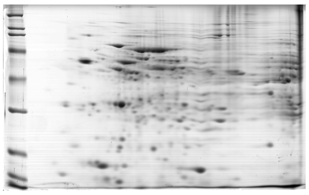
B6-3 100ug

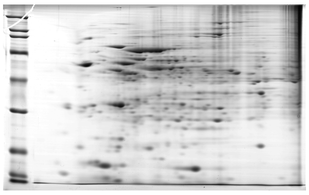
B6-3 100ug

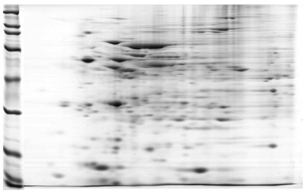
B6-3 100ug

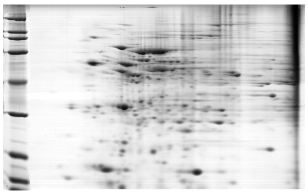
B6-4 100ug

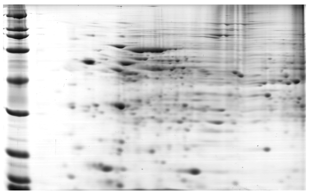
B6-4 100ug

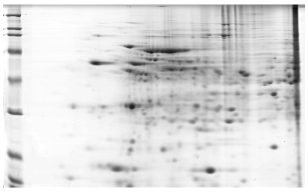
B6-4 100ug

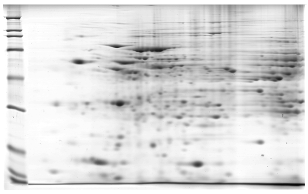
B6-5 100ug

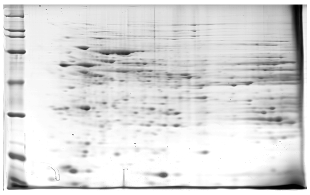
B6-5 100ug

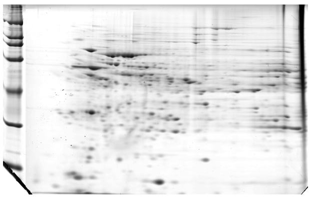
B6-7 100ug

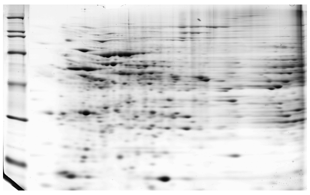
B6-7 100ug

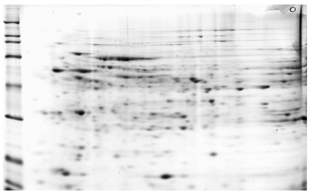
B6-7 100ug

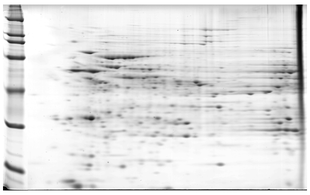
B6-8 100ug

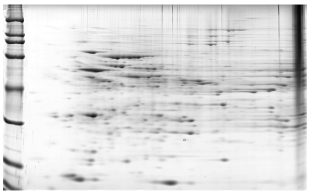
B6-8 100ug

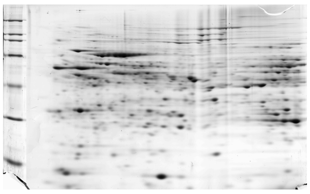
B6-8 100ug

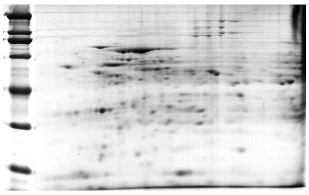
B6-10 100ug

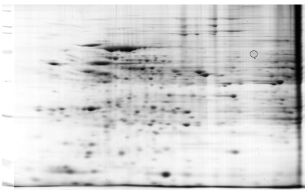
B6-10 100ug

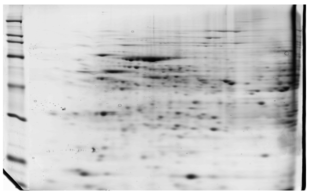
B6-10 100ug

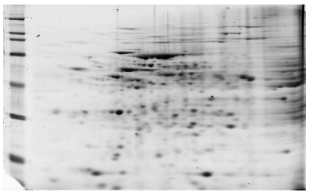
B6-11 100ug

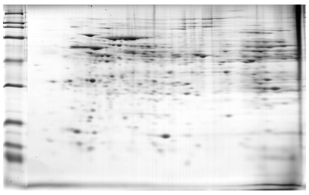
B6-12 100ug

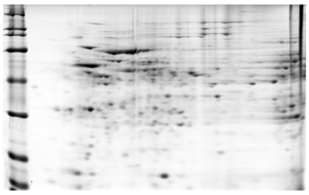
B6-12 100ug

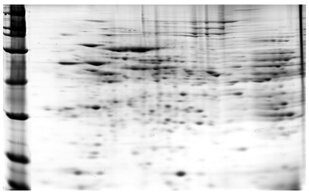
B6-12 100ug

Figure S3. 2D-PAGE replicates of Lp(a) mouse liver

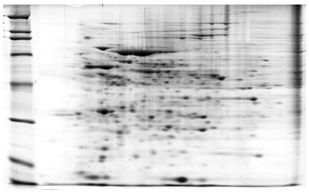
Lp(a)-4 100ug

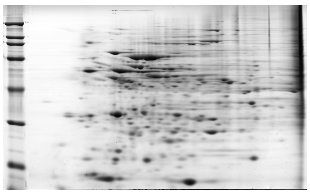
Lp(a)-3 100ug

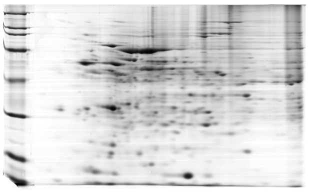
Lp(a)-9 100ug

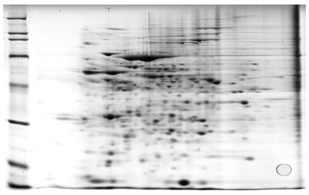
Lp(a)-4 100ug

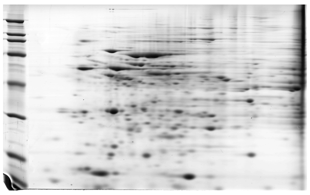
Lp(a)-3 100ug

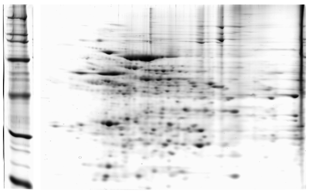
Lp(a)-9 100ug

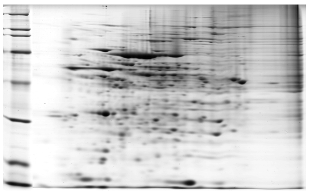
Lp(a)-4 100ug

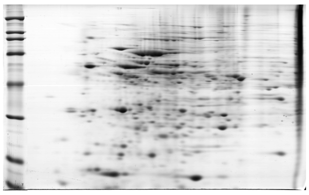
Lp(a)-3 100ug

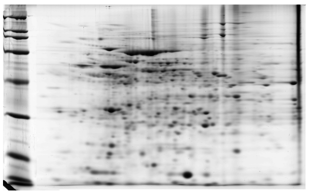
Lp(a)-10 100ug

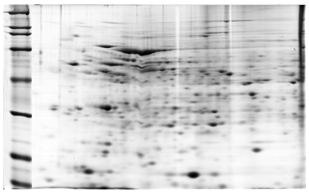
Lp(a)-5 100ug

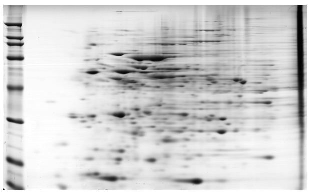
Lp(a)-3 100ug

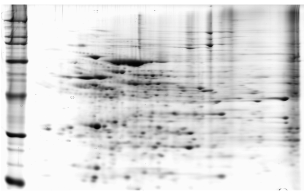
Lp(a)-11 100ug

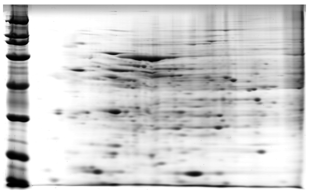
Lp(a)-5 100ug

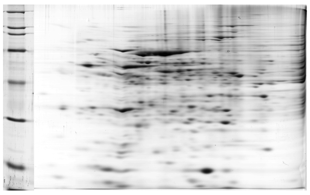
Lp(a)-7 100ug

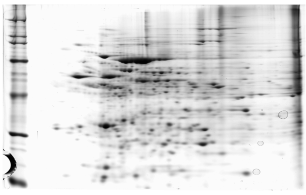
Lp(a)-11 100ug

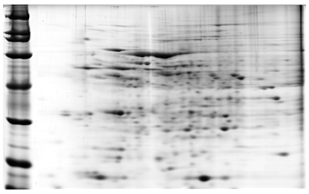
Lp(a)-5 100ug

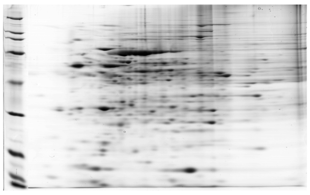
Lp(a)-7 100ug

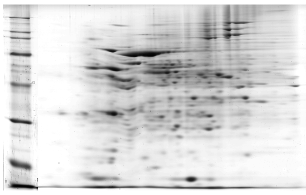
Lp(a)-12 100ug

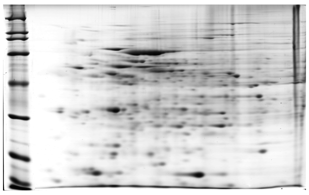
Lp(a)-5 100ug

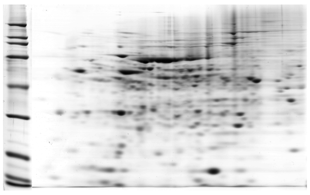
Lp(a)-7 100ug

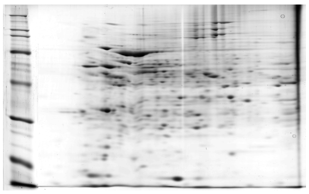
Lp(a)-12 100ug

Table S1. Proteins identified by MALDI-TOF MS/MS from the livers of wildtype and Lp(a) mice on a normal chow diet

Spot number	Identified Protein (Swiss-Prot Accession Number)	Masses Matched (C.I. > 95%)	Three Highest Ranked Peptide Sequences (C.I. > 95%)	Total Ion Score
1	*Park7*<br>Protein DJ-1 (Q99LX0)	3	TQGPYDVVVLPGGNLGAQNLSESPMVK<br>GPGTSFEFALAIVEALVGK<br>GAEEMETVIPVDVMR	291
2	*Prdx6*<br>Peroxiredoxin 6 (O08709)	5	FHDFLGDSWGILFSHPR<br>LIALSIDSVEDHLAWSK<br>LPFPIIDDK	488
3	*Prdx6*<br>Peroxiredoxin 6 (O08709)	10	PGGLLLGDEAPNFEANTTIGR<br>FHDFLGDSWGILFSHPR<br>LIALSIDSVEDHLAWSK	798
4	*Prdx6*<br>Peroxiredoxin 6 (O08709)	10	PGGLLLGDEAPNFEANTTIGR<br>FHDFLGDSWGILFSHPR<br>LIALSIDSVEDHLAWSK	733
5	*Gpx1*<br>Glutathione peroxidase 1 (Q5RJH8)	5	YVRPGGGFEPNFTLFEK<br>FLVGPDGVPVR<br>NDIAWNFEK	305
6	*Sod1*<br>Superoxide dismutase [Cu-Zn] (P08228)	6	VISLSGEHSIIGR<br>GDGPVQGTIHFEQK<br>HVGDLGNVTAGK	435
7	*Sod1*<br>Superoxide dismutase [Cu-Zn] (P08228)	2	VISLSGEHSIIGR<br>KHGGPADEER	169
8	*Acaa2*<br>3-ketoacyl-CoA thiolase, mitochondrial (Q8JZR8)	2	DGTVTAGNASGVSDGAGAVIIASEDAVKKHNFTPLAR<br>TPFGAYGGLLKDFSATDLTEFAAR	229

9	*Echs1*<br>Enoyl-CoA hydratase, mitochondrial (Q8BH95)	1	AQFGQPPEILLGTIPGAGGTQR	133
10	*Apoa4*<br>Apolipoprotein A-IV (P06728)	1	ALVQQLEQFR	111
11	*Apoa1*<br>Apolipoprotein A-I (Q00623)	4	LAELKSNPTLNEYHTR<br>VAPLGAELQESAR<br>WKEDVELYR	358
12	*Fabp4*<br>Fatty acid-binding protein (P04117)	9	LVSSENFDDYMKEVGVGFATR<br>SIITLDGGALVQVQK<br>LGVEFDEITADDRK	922
13	*Serpinc1*<br>Antithrombin III (P32261)	2	AFLEVNEEGSEAAASTSVVITGR<br>LQPLDFKENPEQSR	104
14	*Ywhag*<br>14-3-3 protein gamma (P61982)	2	NVTELNEPLLSNEERNLLSVAYKNVVGAR<br>NLLSVAYKNVVGAR	
15	*Pebp1*<br>Phosphatidylethanolamine-binding protein 1 (P70296)	4	GNDISSGTVLSDYVGSGPPSGTGLHR<br>VDYAGVTVDELGK<br>LYTLVLTDPDAPSR	455
16	*Ca3*<br>Carbonic anhydrase 3 (P16015)	5	YAAELHLVHWNPK<br>QFHLHWGSSDDHGSEHTVDGVK<br>VVFDDTYDR	367
17	*Glo1*<br>Lactoylglutathione lyase (Q9CPU0)	6	GLAFIQDPDGYWIELNPNK<br>FSLYFLAYEDKNDIPK<br>DFLLQQTMLR	435

18	*Uqcrc1*<br>Cytochrome b-c1 complex subunit 1, mitochondrial (Q00896)	3	EVESIGAHLNAYSTR<br>MVLAAAGGVEHQQLLDLAQK<br>TDLTDYLNR	239
19	*Cyb5a*<br>Cytochrome b5 (P56395)	5	EQAGGDATENFEDVGHSTDAR<br>FLEEHPGGEEVLR<br>TYIIGELHPDDR	442
20	*Atp5h*<br>ATP synthase subunit d, mitochondrial (Q9DCX2)	6	IPVPEDKYTALVDQEEKEDVK<br>LASLSEKPPAIDWAYYR<br>ANVAKPGLVDDFEKK	496
21	*Ppa1*<br>Inorganic pyrophosphatase (Q9D819)	4	VIAINVDDPDAANYK<br>DVFHMVVEVPR<br>GQYISPFHDVPIYADK	370
22	*Haao*<br>3-hydroxy anthranilate 3,4-dioxygenase (Q78JT3)	8	DLGTQLAPIIQEFFHSEQYR<br>AQSVALSTQDPAR<br>FANTMGLVIER	555
23	*Pnpo*<br>Pyridoxine-5'-phosphate oxidase (Q91XF0)	2	EAENYFHSRPK<br>FFTNYESR	146
24	*Tpt1*<br>Translationally-controlled tumour protein (P63028)	3	DLISHDELFSDIYK<br>GKLEEQKPER<br>EDGVTPFMIFFK	226
25	*Pdia3*<br>Protein disulfide isomerase A3 (P27773)	8	FISDKDASVVGFFR<br>ALEQFLQEYFDGNLK<br>DLLTAYYDVDYEK	572
26	*Hspd1*<br>60 kDa heat shock protein, mitochondrial (P63038)	9	DMAIATGGAVFGEEGLNLNLEDVQAHDLGK<br>LVQDVANNTNEEAGDGTTTATVLAR<br>ALMLQGVDLLADAVAVTMGPK	904

27	*Pbld2*<br>Phenazine biosynthesis-like domain-containing protein 2 (Q9CXN7)	5	GLILTVKGEPGGQTALYDFYSR<br>KLQPTDDFTQSSR<br>TLDEDAHQDIAR	432
